# BNP but Not s-cTnln Is Associated with Cardioembolic Aetiology and Predicts Short and Long Term Prognosis after Cerebrovascular Events

**DOI:** 10.1371/journal.pone.0102704

**Published:** 2014-07-29

**Authors:** Nicole Nigro, Karin Wildi, Christian Mueller, Philipp Schuetz, Beat Mueller, Felix Fluri, Mirjam Christ-Crain, Mira Katan

**Affiliations:** 1 Department of Endocrinology, University Hospital Basel, Basel, Switzerland; 2 Department of Cardiology, University Hospital Basel, Basel, Switzerland; 3 Medical University Clinic and Division of Endocrinology, Diabetology and Metabolism, Kantonsspital Aarau, Aarau, Switzerland; 4 Department of Neurology, University Hospital Zurich, Zurich, Switzerland; 5 Department of Neurology, University Hospital Basel, Basel, Switzerland; 6 Department of Clinical Research, University Hospital Basel, Basel, Switzerland; University of Glasgow, United Kingdom

## Abstract

**Background:**

We analyzed the prognostic value of b-type natriuretic peptide (BNP) and sensitive cardiac Troponin (s-cTnI) in patients with ischemic stroke or transient ischemic attack (TIA) and their significance in predicting stroke aetiology.

**Methods:**

In a prospectively enrolled cohort we measured BNP and s-cTnI levels upon admission. Primary endpoints were mortality, unfavorable functional outcome and stroke recurrence after 90 days and after 12 months. Secondary endpoint was cardioembolic aetiology.

**Results:**

In 441 patients BNP but not s-cTnI remained an independent predictor for death with an adjusted HR of 1.2 (95% CI 1.1–1.4) after 90 days and 1.2 (95% CI 1.0–1.3) after one year. The comparison of the Area under Receiver Operating Characteristic (AUROC) of model A (age, NIHSS) and model B (age, NIHSS, BNP) showed an improvement in the prediction of mortality (0.85 (95% CI 0.79–0.90) vs. 0.86 (95% CI 0.81–0.92), Log Rank p = 0.004). Furthermore the category free net reclassification improvement (cfNRI) when adding BNP to the multivariate model was 57.5%, p<0.0001. For the prediction of functional outcome or stroke recurrence both markers provided no incremental value. Adding BNP to a model including age, atrial fibrillation and heart failure lead to a higher discriminatory accuracy for identification of cardioembolic stroke than the model without BNP (AUC 0.75 (95% CI 0.70–0.80) vs. AUC 0.79, (95% CI 0.75–0.84), p = 0.008).

**Conclusion:**

BNP is an independent prognostic maker for overall mortality in patients with ischemic stroke or TIA and may improve the diagnostic accuracy to identify cardioembolic aetiology.

**Trial Registration:**

ClinicalTrials.gov NCT00390962

## Introduction

Several prognostic [1] and some aetiological biomarkers have been evaluated in ischemic stroke but so far most failed to show incremental information. Aetiological classification of ischemic stroke and TIA is critical to discern the best treatment for specific secondary prevention. In a European population-based study the highest recurrence rate after stroke or TIA was found among patient with cardioembolic aetiology when compared to all other types of aetiology [2]. Therefore, early identification of cardioembolic aetiology of stroke is crucial.

B-type natriuretic peptide (BNP) is a neurohormone secreted predominantly from the myocardium in response to wall stretch or increased intracardiac pressure [3]–[6]. High BNP levels have been shown to predict atrial fibrillation within the general population as well as in stroke patients [7]. BNP levels were associated with cardioembolic stroke aetiology [8], [9]. Furthermore, high plasma levels of BNP were an independent predictor of short and long-term mortality [10], [11] and of functional outcome at 6 months after ischemic stroke [12], [13]. New published data show that BNP levels are associated with TIA recurrence after a TIA [14]. However, these data base on a small group of patients and need to be verified in a larger cohort. Recently, a meta-analysis found that BNP levels were associated with all cause mortality but not with functional outcome. The incremental prognostic value concerning overall mortality, however, was limited. The authors of this meta-analysis concluded with the remark that further well designed cohort studies are needed to assess the predictive value of BNP in stroke patients [15].

Troponin is a highly sensitive and specific marker of myocardial necrosis and has an important role in the diagnosis of myocardial infarction [16]. Similar to BNP, also elevated s-cTnI has been suggested as marker for increased risk of mortality after an acute stroke [17]–[19]. However data on Troponin in the setting of stroke are more spars. To our knowledge, there are no data on the association of s-cTnI levels and stroke recurrence.

The purpose of this study was, first, to further validate BNP and s-cTnI as prognostic markers after stroke and TIA and, second, to evaluate their ability to identify cardioembolic aetiology.

## Material and Methods

### Study design and setting

The study design of this prospective cohort study has been described in detail elsewhere (ClinicalTrials. gov number, NCT00390962) [20]. Briefly, between November 2006 and November 2007, all consecutive patients (n = 605) presenting at the emergency department of the University Hospital Basel, Switzerland, a 660–bed tertiary care hospital, with a suspected cerebrovascular event were evaluated. All patients presenting with an ischemic stroke or a TIA were included after obtained written informed consent from the patient or the patients' next of kin. We defined initial ischemic stroke according to the World Health Organization criteria as an acute focal neurological deficit lasting longer than 24 hours [21]. TIA was defined accordingly as a transient episode of neurological dysfunction caused by focal brain ischemia with symptoms lasting 24 hours or less. Exclusion criteria were missing informed consent or any diagnosis different from ischemic stroke or TIA (i.e. stroke/TIA mimics)

The Ethics Committee of Basel, Switzerland, approved the trial protocol; informed consent was obtained from all patients.

The protocol for this trial and supporting STROBE Checklist are available as supporting information (see Checklist S1 and Protocol S1).

### Clinical variables

On admission, the following data were collected with a standardized bed-side interview and complete chart review: vital signs, co-morbidities as assessed by the Charlson Comorbidity Index (CCI) adjusted for stroke, medication prior to ischemic stroke or TIA and cardiovascular risk factors (i.e. age, gender, smoking habits, history of hypercholesterolemia, hypertension, diabetes mellitus, previous TIA or stroke, positive family history for myocardial infarction, stroke or TIA and history of coronary heart disease).

Severity of stroke was prospectively assessed on admission by a neurologist certified in the use of the National Institute of Health Stroke Scale (NIHSS) [22]. Risk stratification for patients with a TIA was performed according to the ABCD2-score [23].

Stroke aetiology was determined according to the criteria of the TOAST classification [24], which distinguishes large-artery atherosclerosis, cardioembolism, small-artery occlusion, other aetiology and undetermined aetiology. All patients underwent the necessary diagnostic work-up to classify aetiology according to the TOAST criteria [24] such as standard 12-lead electrocardiography and at least 24-hour electrocardiography, echocardiography and neurosonographic study of the extra- and intracranial arteries as well as routine laboratory testing.

The 90 days and the 12 months follow-up was performed using structured telephone interviews from a blinded stroke physician to assess mortality from any cause, functional outcome using the modified Rankin Scale (mRS) and recurrent cerebrovascular events.

### Blood sampling

In all patients blood samples were obtained on admission within 72 hours from symptom onset. BNP and sensitive cardiac Troponin T were measured in a batch analysis blinded to clinicians. After centrifugation, samples were frozen at −70°C until they were assayed in a blinded fashion in a dedicated core laboratory. S-cTnI was measured in EDTA-plasma using the recently refined s-cTnI assay (Abbott-Architect, Abbott Laboratories, Abbott Park, IL, USA) [25]–[27]. For this test, a limit of detection (LoD) of 0.01 µg/L, a 99^th^-percentile cut-off of 0.028 µg/L and a coefficient of variation (CV) of <10% of 0.032 µg/L have been reported by the manufacturer. Three s-cTnI strata were predefined: below the limit of detection (<0.01 µg/L, undetectable), detectable but still in the normal range (0.01–0.027 µg/L), and increased (≥0.028 µg/L, above the 99th percentile of healthy individuals). The concentration of BNP was measured in EDTA-plasma using the Architect BNP assay (Abbott Laboratories, Abbott Park, IL, USA). The analytical range as reported by the manufacturer extends from 15 to 20'000 pg/ml for the AxSYM assay.

### Endpoints

The primary endpoints of this analysis were a) mortality within 90 days and 1 year in stroke as well as TIA patients b), functional outcome after 90 days and at 1 year in stroke patients, c) stroke or TIA recurrence within 90 days.

Patients and or their caregivers were questioned by structured telephone interviews from blinded stroke physicians at 3 months and 1 year after the index event to assess mortality from any cause, functional outcome using the modified Rankin Scale (mRS) and recurrent cerebrovascular events. Poor outcome was defined as mRS>2 points. A recurrent cerebrovascular event was defined as an as an acute ischemic lesion in the brain not attributed to infection, tumor, demyelination or a degenerative neurologic disease but due to an occlusive vascular disorder. Further criteria were rapid onset of a focal neurological deficit occurring for at least 24 hours in conjunction with brain imaging consistent with acute ischemic stroke. The CT or MRI may either show a new infarct or no change from the study performed at entry, i.e. the diagnosis is clinical and does not require CT/MRI confirmation.

The secondary endpoint was cardioembolic aetiology of stroke diagnosed during hospitalization according to TOAST criteria [24] by stroke physicians, which were blinded to BNP and Troponin measurements.

### Neuroimaging

CCT was performed in all patients on admission mainly to exclude intracranial hemorrhage. Additionally MRI was performed in 197 (45%) patients on a clinical 1.5 T MR Avant system (SIEMENS, Erlangen, Germany) using a stroke protocol, including T1-, T2-, and diffusion-weighted (DWI) sequences, as well as apparent diffusion coefficient (ADC) maps and a MR angiography. Lesions were ranked into three sizes to represent typical stroke patterns: (1) small lesion with a volume of less than 10 ml, (2) medium lesion of 10 to 100 ml and (3) large lesion with a volume of more than 100 ml [28].

### Statistical analysis

Discrete variables are expressed as frequency (percentage), continuous Gaussian variables as means with standard deviation SD and non-Gaussian variables as medians with interquartile ranges [IQR]. Comparisons between groups were made using chi-square test, Mann-Whitney U test and Kruskal-Wallis test as appropriate.

Univariate Cox Proportional Hazard Analysis was utilized to compute hazard ratios and 95% confidence intervals (CI) for potential predictors of death during the follow-up period. All significant variables with a p-value <0.001 (i.e. BNP levels, age, NIHSS, and lesion size) were tested in a multivariate model using the Forward Stepwise Method to avoid over fitting of the model.

To estimate association of BNP and Troponin with functional outcome and re-events, we used logistic regression models. In this analysis we tested all significant variables in the multivariate analysis with a p-value <0.05 (i.e. BNP levels, age, NIHSS, Charlson Comorbidity Index, CRP, heart failure, atrial fibrillation and lesion size). Due to a greater number of endpoints the risk of over fitting was minimal. Receiver operating characteristic (ROC) curves were constructed for different models to assess the discriminative value in terms of sensitivity and specificity for mortality, outcome and aetiology. Comparison was made using the DeLong test [29] and likelihood ratios for nested models as appropriate. Furthermore we calculated the continuous (category free) net reclassification improvement (NRI) [30] for the primary endpoint, i.e. mortality to assess the incremental value of BNP over the multivariate prognostic model, which included the following variables: age and NIHSS. For the secondary endpoint, i.e. cardioembolic aetiology, the NRI was calculated to assess the incremental value of BNP over the multivariate model including age, atrial fibrillation and heart failure. Analyses were performed using SPSS (SPSS 21, Chicago, IL, USA) and MedCalc (MedCalc 10.4, Mariakerke, Belgium), p-values less than 0.05 were considered to indicate statistical significance.

## Results

### Patients

**Figure 1:**
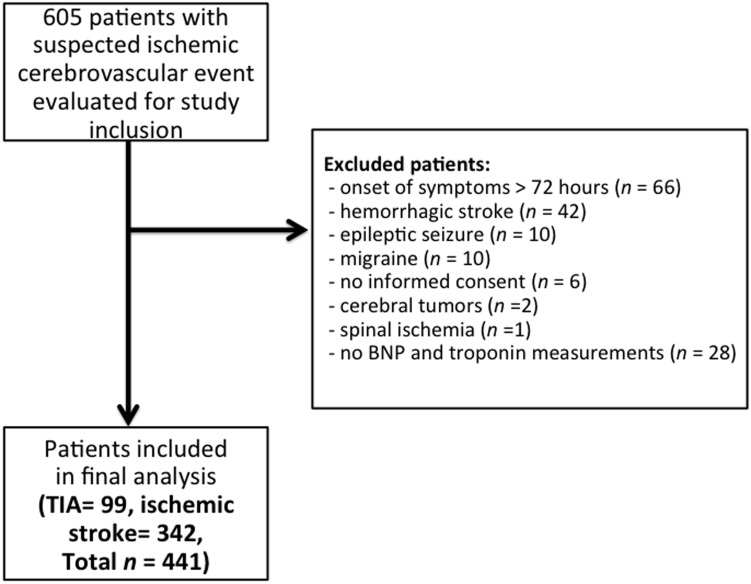
Study flow chart.

From 605 screened patients, 469 patients were eligible for our study and 466 completed follow up after 90 days or one year, respectively. In 362 of the 469 patients ischemic stroke was diagnosed, while 107 out of the 469 had a TIA. 28 patients were excluded from the final analysis because BNP and s-cTnI levels were missing (**Figure 1**).

Baseline demographic and cardiovascular risk factors including NIHSS and lesion size were not different when comparing patients with and without available measurements.

### Baseline characteristics

The median age was 74.6 (IQR 62.6–81.9) years and 42% were women. On admission, the median NIHSS in stroke patients was 5 points (IQR 2–10) and median ABCD2 Score in TIA patients was 3 (IQR 3–5). After 90 days, overall 42 patients had died and the mortality rate was thus 9.5%. After the one-year follow-up, 67 patients had died and the mortality rate was 15.2%. After 90 days 138 (31.3%) of patients with ischemic stroke had an unfavorable outcome after one year 141 patients (32.0%) of 336 patients with a completed follow up had an unfavorable outcome (mRS>2). Re-events were identified in 27 (6.1%) patients with ischemic stroke or TIA.

The principal baseline characteristics of all patients are provided in **Table 1**. There were no significant differences between stroke and TIA patients concerning demographic and risk factor variables besides the etiologies. In patients with TIA we found more undetermined etiologies (50.1%) than in patients with an ischemic stroke (24.0%) (P<0.0001) and in patients with an ischemic stroke we found more patients with a cardioembolic source (37.4%) than in patients with a TIA (8%) (p<0.0001).

**Table 1:** Baseline characteristics of patients with ischemic stroke and transient ischemic attack.

Baseline characteristics (n = 441)	
Demographic findings:	
Cerebrovascular re-events, n (%)	27 (6.1)
Death from any cause, n (%)	67 (15.2)
Age, in years	74.6 (62.6–81.9)
Female sex, n (%)	185 (42)
NIHSS at admission, points	5 (2–10)
Charlson score at admission, points	1 (0–2)
ABCD2 score for TIA severity	4 (3–5)
Clinical findings:	
Heart rate, in bpm	76 (67–88)
Systolic blood pressure, in mmHg	161 (±29)
Diastolic blood pressure, in mmHg	90 (80–100)
Body temperature, in °C	37.0 (±0.7)
Laboratory findings:	
BNP, in pg/ml	81.9 (35.0–203.3)
Troponin I, in ug/l	0.003 (0.000–0.012)
Glucose, in mmol/L	6.1 (5.3–7.3)
Stroke aetiology, n (%):	
Small vessel occlusive	81 (18.4)
Large vessel occlusive	79 (17.9)
Cardioembolic	143 (32.4)
Other	19 (4.3)
Undetermined aetiology	119 (27.0)
Cardiovascular risk factors, n (%):	
Hypertension	332 (75.3)
Atrial fibrillation	83 (18.8)
Heart failure	60 (13.6)
Smoking history	154 (34.9)
Dyslipidemia	121 (27.4)
Diabetes mellitus	81 (18.4)
Coronary heart disease	104 (23.6)
Prior TIA or stroke	114 (25.9)
Family history of AMI or stroke	140 (31.7)
Functional outcome (only for stroke patients, n = 344), n (%):	
Bad outcome after 90 days	138 (31.3)
Bad outcome after 1 year	141 (32.0)
Clinical stroke syndrome, n (%):	
TACS	38 (8.6)
PACS	155 (35.1)
LACS	68 (15.4)
POCS	80 (18.1)

Discrete variables are expressed as frequency (percentage) and continuous Gaussian variables as means with SD and non-Gaussian variables as medians with interquartile ranges [IQR].

NIHSS =  National Institutes of Health Stroke Scale; AMI =  acute myocardial infarction; TACS =  total anterior circulation syndrome; PACS =  partial anterior circulation syndrome; LACS =  lacunar syndrome; POCS =  posterior circulation syndrome.

## Main results

### Cardiac biomarkers and mortality

Median BNP levels in patients with ischemic stroke or TIA were 81.9 (IQR 35.0–203.3) pg/ml. BNP levels were higher in patients who died (n = 46) as compared to patients who survived (n = 395) (309.9 [IQR 162.2–575.8] vs. 69.6 [IQR 31.4–165.3] pg/ml, p<0.0001) after 90 days. These results were similar for death after one year (279.0 [IQR 98.4–493.4] vs. 67.8 [IQR 331.4–153.8] pg/ml, p<0.0001).

Median s-cTnI levels in patients with ischemic stroke or TIA were 0.003 (IQR 0.000–0.012) µg/L. 310 patients had s-cTnI level below the detection limit (<0.01 µg/L), 65 patients in the normal range (0.01–0.027 µg/L), and 66 patients had an increased s-cTnI level (≥0.028 µg/L). Similarly as for BNP, patients who died showed higher s-cTnI levels as compared to patients who survived after 90 days and one year (0.015 [IQR 0.0045–0.076] vs. 0.0030 [IQR 0.000–0.0100] µg/L, p<0.0001) and (0.011 [IQR 0.003–0.055] vs. 0.003 [IQR 0.000–0.010] µg/L, p<0.0001).

In multivariate cox regression analysis BNP but not s-cTnI remained an independent predictor for death within 90 days with a hazard ratio (per 100pg/ml unit's increase) of 1.2 (95% CI 1.1–1.4). The other independent predictors for death after 90 days were age and lesion size (see **Table 2**). Also for long term mortality (within 1 year) only BNP levels (HR 1.2 (95% CI 1.0–1.3), age (per 10 years increase) (HR 3.43 (95% CI 1.6–7.3) and lesion size (HR 1.2 (95% CI 1.0–1.3) remained independent predictors (see **Table 3**). In a multivariate analysis including coronary heart disease, atrial fibrillation and heart failure instead of the CCI, the hazard ratio of BNP remained similar for 90 days and for one year (HR 1.2 (95% CI 1.0–1.3) and (HR 1.2 (95% CI 1.0–1.3), respectively) while the other parameters did not remain significant in the multivariate model.

**Table 2:** Cox regression analysis for mortality after 90 days for all patients (n = 441).

	Univariate Analysis		Multivariate Analysis	
Variables	Hazard ratio (95% Cl)	p-value	Hazard ratio (95% Cl)	p-value
BNP (per 100 pg/ml)	1.2 (1.1–1.2)	<0.001	1.2 (1.1–1.4)	0.007
Troponin I at presentation (per 100 µg/l)	1.0 (1.0–1.0)	0.086		
Age (per 10 years)	2.2 (1.5–3.3)	<0.001	3.2 (1.4–7.6)	0.007
Female sex	1.0 (0.5–1.9)	0.965		
Systolic blood pressure (per 10 mmHg)	0.9 (0.8–1.0)	0.045		
Temperature (per °C)	0.7 (0.4–1.2)	0.23		
CRP (per 10 mg/dl)	1.1 (1.0–1.1)	0.001		
Glucose (per mmol/l)	1.1 (0.9–1.2)	0.48		
NIHSS	1.1 (1.1–1.2)	<0.001	1.1 (1.0–1.2)	0.2
Charlson Score	1.2 (1.0–1.4)	0.02		
History of				
- Hypertension	1.3 (0.6–2.9)	0.47		
- Coronary heart disease	2.0 (1.1–3.9)	0.03		
- Heart failure	2.7 (1.3–5.3)	0.006		
- Atrial fibrillation	3.0 (1.6–5.7)	0.001		
- Dyslipidemia	1.1 (0.6–2.2)	0.75		
- Family history of stroke and/or AMI	0.8 (0.4–1.6)	0.49		
- Diabetes mellitus	1.3 (0.6–2.7)	0.51		
- Smoking	0.9 (0.4–1.7)	0.65		
- Prior stroke	0.7 (0.3–1.5)	0.39		
Cardioembolic aetiology	1.5 (0.8–2.9)	0.2		
Lesion size (per 10 ml)	1.2 (1.1–1.2)	<0.001	1.2 (1.0–1.3)	0.01

CI =  confidence interval; CRP =  C-reactive protein; NIHSS =  National Institutes of Health Stroke Scale; AMI =  acute myocardial infarction.

**Table 3:** Cox regression analysis for mortality after one year for all patients (n = 441).

	Univariate Analysis		Multivariate Analysis	
Variables	Hazard ratio (95% Cl)	p-value	Hazard ratio (95% Cl)	p-value
BNP (per 100 pg/ml)	1.2 (1.1–1.2)	<0.001	1.2 (1.0–1.3)	0.02
Troponin I at presentation (per 100 µg/l)	1.0 (1.0–1.0)	0.19		
Age (per 10 years)	2.3 (1.4–3.0)	<0.001	3.4 (1.6–7.3)	0.001
Female sex	1.3 (0.8–2.1)	0.39		
Systolic blood pressure (per 10 mmHg)	0.9 (0.6–1.0)	0.12		
Temperature (per °C)	0.8 (0.5–1.2)	0.25		
CRP (per 10 mg/dl)	1.1 (1.0–1.1)	0.023		
Glucose (per mmol/l)	1.1 (1.0–1.2)	0.18		
NIHSS (per point)	1.1 (1.1–1.2)	<0.001	1.1 (1.0–1.2)	0.2
Charlson Score (per point)	1.3 (1.1–1.4)	<0.001	1.0 (0.8–1.6)	0.6
History of				
- Hypertension	1.1 (0.6–2.0)	0.71		
- Coronary heart disease	1.6 (0.9–2.8)	0.12		
- Heart failure	2.6 (1.5–4.5)	0.001		
- Atrial fibrillation	3.3 (2.0–5.5)	<0.001		
- Dyslipidemia	0.9 (0.5–1.6)	0.79		
- Family history of stroke and/or AMI	0.80(0.5–1.4)	0.42		
- Diabetes mellitus	1.0 (0.5–1.9)	0.95		
- Smoking	0.6 (0.3–1.0)	0.063		
- Prior stroke	0.9 (0.5–1.7)	0.8		
Cardioembolic aetiology	1.6 (0.9–2.6)	0.1		
Lesion size (per 10 ml)	1.2 (1.1–1.2)	<0.001	1.2 (1.0–1.3)	0.01

CI =  confidence interval; CRP =  C-reactive protein; NIHSS =  National Institutes of Health Stroke Scale; AMI =  acute myocardial infarction.

BNP was predictive also across all subgroups stratified by presence of atrial fibrillation (HR of BNP among pat with AF 1.16, p = 0.01 after 90 days and HR 1.14, p = 0.005 after one year respectively, without AF 1.19, p<0.001 after 90 days and HR 1.18, p<0.001 after one year respectively) or heart insufficiency (HR of BNP in patients with heart insufficiency 1.12, p<0.001 after 90 days and HR 1.12, p<0.001 after one year respectively, HR of BNP in patients without heart insufficiency 1.24, p<0.001 after 90 days and HR 1.16, p<0.001 after one year respectively), thus we found no relevant effect modification.

Moreover the AUC of BNP to predict mortality after 90 days was 0.81 (95% CI 0.74–0.83) and 0.75 (95% CI 0.68–0.81) after one year respectively. The comparison of ROC curves of model A (i.e. the validated prognostic model of Koenig et al [31] including age and the NIHSS) and model B (age, NIHSS, BNP) revealed an improvement in the prediction of mortality (AUC 0.85 (95% CI 0.79–0.90) vs. AUC 0.86 (95% CI 0.81–0.92), p = 0.004). The category free net reclassification improvement (cfNRI) when adding BNP to the best prognostic model was 57.5%, p<0.0001.

### Cardiac biomarkers and functional outcome

For functional outcome only patients with an ischemic stroke (n = 344) were considered. BNP levels in patients with an unfavorable outcome after 90 days and after one year were significantly higher than those in patients with a favorable outcome (150.0 [IQR 57.3–346.5] vs. 59.9 [IQR 30.2–145.2] pg/ml, (p<0.0001) for 90 days and 154.1 [IQR 61.5–346.5] vs.59.9 [IQR 30.2–145.2] pg/mL, (p<0.0001) for one year). S-cTnI levels were higher in patients with an unfavorable outcome as compared to patients with a good outcome (0.008 [IQR 0.001–0.0265] vs. 0.003 [IQR 0.000–0.008] µg/L, (p<0.0001) for 90 days (see **Table 4**) and 0.008 [IQR 0.001–0.0285] vs. 0.003 [IQR 0.000–0.0105] µg/L, (p = 0.0008) for 1 year) (see **Table 5**). However, after adjustment for all other significant outcome predictors, the only independent outcome predictors were the NIHSS, age and lesion size (see **Table 4** and **Table 5**).

**Table 4:** Logistic regression analysis for functional outcome after 90 days for ischemic stroke patients (n = 342).

	Univariate Analysis		Multivariate Analysis			
Variables	OR (95% Cl)	p-value		OR (95% Cl)	p-value	
BNP (per 100 pg/ml)	1.2 (1.1–1.3)	<0.001		1.1 (0.9–1.3)	0.26	
Troponin I at presentation (per 10 ug/l)	1.0 (1.0–1.0)	0.79				
Age	1.1 (1.0–1.1)	<0.001		1.0 (1.0–1.1)	0.07	
Female sex	1.6 (1.1–2.5)	0.029		1.0 (0.4–1.1)	0.95	
NIHSS (per point)	1.2 (1.1–1.2)	<0.001		1.1 (1.0–1.5)	0.046	
Charlson Score (per point)	1.3 (1.1–1.6)	<0.001		1.2 (0.8–1.6)	0.34	
Systolic blood pressure (per 10 mmHg)	1.0 (0.9–1.0)	0.29				
Temperature (per °C)	0.8 (0.6–1.2)	0.33				
Heart beat (per 10/min)	1.1 (1.0–1.3)	0.22				
CRP (per 10 mg/dl)	1.1 (1.0–1.2)	0.02		1.0 (0.9–1–2)	0.58	
Glucose (per mmol/l)	1.1 (1.0–1.2)	0.21				
History of						
- Hypertension	1.5 (0.9–2.6)	0.11				
- Coronary heart disease	1.3 (0.8–2.1)	0.37				
- Heart failure	2.8 (1.5–5.2)	0.001		1.5 (0.4–5.7)	0.56	
- Atrial fibrillation	1.9 (1.1–3.2)	0.02		1.1 (0.4–3.0)	0.82	
- Dyslipidemia	0.8 (0.5–1.4)	0.43				
- Family history of stroke and/or AMI	0.8 (0.5–1.3)	0.29				
- Diabetes mellitus	1.2 (0.7–2.0)	0.62				
- Smoking	0.8 (0.5–1.2)	0.22				
Lesion size (per 10 ml)	1.2 (1.1–1.3)	<0.001		1.2 (1.0–1.3)	0.02	

OR =  odds ratio; CI =  confidence interval; CRP =  C-reactive protein; NIHSS =  National Institutes of Health Stroke Scale; AMI =  acute myocardial infarction.

**Table 5:** Logistic regression analysis for functional outcome after one year for ischemic stroke patients (n = 342).

	Univariate Analysis		Multivariate Analysis	
	OR (95% Cl)	p-value	OR (95% Cl)	p-value
BNP (per 100 pg/ml)	1.2 (1.1–1.4)	<0.001	1.1 (1.0–1.3)	0.14
Troponin I at presentation (per 10 ug/l)	1.0 (1.0–1.0)	0.88		
Age	1.1 (1.1–1.1)	<0.001	1.1 (1.0–1.1)	0.01
Female sex	1.6 (1.0–2.4)	0.05		
NIHSS (per point)	1.1 (1.1–1.2)	<0.001	1.1 (1.0–1.2)	0.01
Charlson Score (per point)	1.4 (1.2–1.6)	<0.001	1.2 (0.9–1.5)	0.29
Systolic blood pressure (per 10 mmHg)	1.0 (0.9–1.0)	0.21		
Temperature (per °C)	0.9 (0.6–1.3)	0.44		
Heart beat (per 10/min)	1.1 (1.0–1.3)	0.14		
CRP (per 10 mg/dl)	1.0 (1.0–1.0)	0.39		
Glucose (per mmol/l)	1.0 (0.9–1.1)	0.86		
History of				
- Hypertension	1.2 (0.7–2.0)	0.46		
- Coronary heart disease	1.2 (0.7–2.0)	0.5		
- Heart failure	2.5 (1.3–4.7)	0.004	1.7 (0.5–5.6)	0.41
- Atrial fibrillation	2.0 (1.2–3.4)	0.012	1.1 (0.4–2.7)	0.9
- Dyslipidemia	0.7 (0.4–1.2)	0.21		
- Family history of stroke and/or AMI	0.5 (0.3–0.9)	0.11		
- Diabetes mellitus	1.4 (0.8–2.4)	0.27		
- Smoking	0.7 (0.4–1.0)	0.07		
Lesion size (per 10 ml)	1.2 (1.1–1.4)	<0.001	1.2 (1.1–1.4)	0.002

OR =  odds ratio; CI =  confidence interval; CRP =  C-reactive protein; NIHSS =  National Institutes of Health Stroke Scale; AMI =  acute myocardial infarction.

### Cardiac biomarkers and cerebrovascular recurrence

BNP levels in patients with a cerebrovascular re-event were similar as compared to patients without a re-event (123.3 [IQR 29.4–207.3] vs. 70.4 [IQR 32.9–165.3] pg/ml, p = 0.499).

The same was true for s-cTnI levels (patients with re-event: 0.003 [IQR 0.000–0.010] vs. patients without re-event: 0.004 [IQR 0.000–0.013] µg/L, p = 0.705).

### Cardiac biomarkers and cardioembolic aetiology

The aetiology for ischemic stroke or TIA was undetermined in 144 patients (33%), 144 patients (33%) had cardioembolic aetiology, 76 patients (17%) had large arteriosclerosis, 61 patients (14%) had a small arteriocclusion and 16 patients (4%) had other etiologies.

Median BNP levels were higher in patients with a cardioembolic stroke or TIA compared to other etiologies (179.5 [IQR 72.5–367.2] vs. 57.2 [IQR 27.5–128.2] pg/ml, p<0.0001). BNP levels showed an AUC of 0.73 (95% CI 0.68–0.78) to predict a cardioembolic source of ischemic stroke or a TIA and thus had a higher predictive value as compared with age (AUC 0.57 (95% CI 0.51–0.62), p<0.001). The combination of BNP with age, the history of or newly detected atrial fibrillation and heart failure in comparison of a nested model had a higher discriminatory accuracy than age, atrial fibrillation and heart failure alone (AUC 0.79, (95%CI 0.75–0.84) vs. AUC 0.75 (95% CI 0.70–0.80, p = 0.008 for comparison). The combination of BNP with the multivariate model led to a category-free net reclassification improvement (cfNRI) of 48.5% (p<0.001) for cardioembolic aetiology.

Patients with a cardioembolic source of ischemic stroke had the highest s-cTnI levels compared to the other etiologies (0.007 [IQR 0.001–0.030] vs. 0.002 [IQR 0.000–0.008] µg/L, p<0.0001). The AUC of s-cTnI to predict a cardioembolic source was 0.62 (95% CI 0.56–0.68).

## Discussion

In this prospective study we evaluated both, BNP and s-cTnI levels, in a large cohort of ischemic stroke and TIA patients for prediction of mortality, functional outcome and re-events after 90 days and 1 year as well as for stroke aetiology.

Our data show the following main findings: First, BNP and s-cTnI levels on admission were elevated in patients with ischemic stroke or TIA who had died or had an unfavorable outcome 90 days and one year after the event. After adjustment for other risk factors BNP but not s-cTnI was an independent predictor of death but not of functional outcome or recurrence. Second, BNP improved the identification of patients with cardioembolic strokes or TIAs when compared to clinical information on admission alone (i.e. age, CHF and the history of atrial fibrillation).

Our data showing an independent association of BNP with mortality is mostly in line with findings in the literature. Studies reported high levels of natriuretic peptides and an association with mortality in acute stroke patients at admission [11], during hospitalization [10] and within 6 months [13] after stroke and a recent meta-analysis concluded that natriuretic peptides are independent predictors for all cause mortality after stroke [15]. However a recent study found no incremental value of BNP for the prediction of mortality compared to clinical variables such as age and NIHSS [11]. This discrepant finding might be due to methodological differences. The cohort included hemorrhages and the primary endpoint was mortality or neurological worsening in the stroke unit, thus within the first days after stroke. There was no assessment of several potentially influencing medical comorbidities or of stroke aetiology. In contrast, in our study we assessed only ischemic stroke and TIA patients and the primary endpoint was mortality within 90 days and 1 year. Our data contained more moderate strokes and thus our cohort had a lower level of NIHSS at admission. We took into account stroke aetiology and several medical comorbidities which are known to affect mortality and functional outcome in stroke patients [32]. Importantly, we adjusted for renal insufficiency (within the CCI), which is known to affect BNP levels [33].

Data regarding the predictive value of BNP for functional outcome in patients after a cerebrovascular event are more controversial. Some studies found an association with functional outcome [13], [34], other studies showed that especially after adjustment in multivariate analyses BNP did not remain an independent prognostic marker for functional outcome [11], [35]. This is in line with our results. It is biologically plausible since functional outcome is probably more prominently influenced by other factors such as brain plasticity. On the other hand, hemodynamic changes and underlying cardiac disease are major contributors to mortality after stroke [36].

Data supporting the association of natriuretic peptides with cardioembolic stroke cumulate. Elevated BNP levels in acute cerebral infarction were associated with cardioembolic aetiology in several studies [8], [37]. However, the incremental value of BNP when added to clinical variables, which are known to be associated with cardioembolic events such as age, has not been evaluated in these studies. Our data show that the combination of BNP with age and atrial fibrillation (i.e. history of or a newly diagnosed atrial fibrillation) had a higher discriminatory accuracy in comparison to the same model without BNP. BNP may therefore have a role in risk stratification predicting mortality on one hand and may support early specific implementation of secondary prevention such as oral anticoagulants on the other hand.

Midregional proANP (MR-proANP), another natriuretic peptide, was assessed for its predictive role concerning mortality, functional outcome and cardioembolic aetiology in patients with an ischemic stroke but not TIA [38]. As BNP, MR- proANP was an independent predictor of mortality after 90 days and improved the prognostic value of the NIHSS. Moreover it was also associated with cardioembolic aetiology improving the diagnostic accuracy of already known clinical information, such as age, known heart failure, and known AF on admission. However, BNP compared to MRproANP is more widely available and clinically established in most countries and physicians are already familiar with it. In this study we evaluated BNP with further statistical methods such as the cfNRI and found a significant improvement in the prediction even if added to models including not only the NIHSS but all significant predictors. However a direct comparison is needed in the future to establish which natriuretic peptide is better for risk classification and identification of cardioembolic strokes. In our cohort, s-cTnI was not an independent marker for all three endpoints. Recently higher Troponin T levels were found in older patients with a more severe ischemic stroke and with comorbidities such as heart failure and/or renal insufficiency [39]. Another study showed that elevated levels of high sensitive Troponin T are frequently present in patients with acute ischemic stroke but, in accordance to our results, did not provide additional prognostic information in these subjects [40]. Interestingly s-cTnI did also not provide additional information for the identification of cardioembolic aetiology in our study. It may be that s-cTnI is too specific for myocardial infarction; it is therefore possible that by measuring s-cTnI levels patients with underlying cardiac disease not directly due to myocardial damage are missed.

To our knowledge, this is the first study that assesses BNP and s-cTnI for prediction of stroke recurrence. Our results show that neither BNP nor Troponin levels were associated with a re-event after ischemic stroke or TIA. Possibly, these markers have no role in stroke recurrence. Alternatively, our study was underpowered to find a significant association with recurrence. Nevertheless our findings suggest that even if there is an association it is of rather moderate clinical relevance.

The following limitations of our study must be taken into account. First, this was a single center study and further evaluation and validation in a larger cohort of patients should be performed. Second, etiological classification is difficult and a large proportion of patients (especially in the TIA group) remained in the undetermined category, however no patient was classified in to this category due to incomplete diagnostic evaluation. Moreover misclassification might have occurred since at the time of patient recruitment we performed mainly 24 hours ECG monitoring but more recent data suggest that longer monitoring increases the identification of intermittent atrial fibrillation substantially [41], [42]. It is therefore possible that we missed some cardioembolic stroke patients. However, this potential misclassification would lead rather to a bias towards the null thus underestimating the positive association. Third, biomarker measurement was performed within a rather large time frame (i.e. within 72 hours after the event). However in 398 patients (90%) blood was drawn within 24 hours of the event, thus it is unlikely that the observed association is only driven by later measurements. Moreover the predictive value remained similar when analyzing BNP levels stratified by time since symptom onset (i.e. 0–3 h, 3–12 h, 12–24 h and 24–72 h) (data not shown). Finally, the lack of association of cardiac biomarkers with stroke recurrence may be due to the relative small number of re-events and thus limited power to find a significant association.

In conclusion BNP but not s-cTnI is an independent marker with incremental value for overall mortality prediction but not for the prediction of functional outcome and re-events in patients with ischemic stroke or TIA. Furthermore BNP levels contribute additional information to prior used clinical variables in identifying cardioembolic stroke aetiology. BNP may therefore be helpful for risk assessment and earlier identification of patients, which may benefit from oral anticoagulation compared to antiplatelet therapy. However, further studies are needed to specifically validate the magnitude of the additional prognostic information, and to evaluate if this information ultimately leads to an improved patient care.

## Supporting Information

Checklist S1 **STROBE checklist.**
(DOC)

Protocol S1 **Trial protocol.**
(DOC)

## References

[1] Katan M, Elkind MS (2011) Inflammatory and neuroendocrine biomarkers of prognosis after ischemic stroke. Expert Rev Neurother 11: 225–239. 21306210 10.1586/ern.10.200

[2] Kolominsky-Rabas PL, Weber M, Gefeller O, Neundoerfer B, Heuschmann PU (2001) Epidemiology of ischemic stroke subtypes according to TOAST criteria: incidence, recurrence, and long-term survival in ischemic stroke subtypes: a population-based study. Stroke 32: 2735–2740. 11739965 10.1161/hs1201.100209

[3] Davis M, Espiner E, Richards G, Billings J, Town I, et al (1994) Plasma brain natriuretic peptide in assessment of acute dyspnoea. Lancet 343: 440–444. 7905953 10.1016/s0140-6736(94)92690-5

[4] Maisel AS, Krishnaswamy P, Nowak RM, McCord J, Hollander JE, et al (2002) Rapid measurement of B-type natriuretic peptide in the emergency diagnosis of heart failure. N Engl J Med 347: 161–167. 12124404 10.1056/NEJMoa020233

[5] Lainchbury JG, Campbell E, Frampton CM, Yandle TG, Nicholls MG, et al (2003) Brain natriuretic peptide and n-terminal brain natriuretic peptide in the diagnosis of heart failure in patients with acute shortness of breath. J Am Coll Cardiol 42: 728–735. 12932611 10.1016/s0735-1097(03)00787-3

[6] Wright SP, Doughty RN, Pearl A, Gamble GD, Whalley GA, et al (2003) Plasma amino-terminal pro-brain natriuretic peptide and accuracy of heart-failure diagnosis in primary care: a randomized, controlled trial. J Am Coll Cardiol 42: 1793–1800. 14642690 10.1016/j.jacc.2003.05.011

[7] Schnabel RB, Larson MG, Yamamoto JF, Sullivan LM, Pencina MJ, et al (2010) Relations of biomarkers of distinct pathophysiological pathways and atrial fibrillation incidence in the community. Circulation 121: 200–207. 20048208 10.1161/CIRCULATIONAHA.109.882241 PMC3224826

[8] Montaner J, Perea-Gainza M, Delgado P, Ribo M, Chacon P, et al (2008) Etiologic diagnosis of ischemic stroke subtypes with plasma biomarkers. Stroke 39: 2280–2287. 18535284 10.1161/STROKEAHA.107.505354

[9] Shibazaki K, Kimura K, Iguchi Y, Okada Y, Inoue T (2009) Plasma brain natriuretic peptide can be a biological marker to distinguish cardioembolic stroke from other stroke types in acute ischemic stroke. Intern Med 48: 259–264. 19252345 10.2169/internalmedicine.48.1475

[10] Shibazaki K, Kimura K, Iguchi Y, Aoki J, Sakai K, et al (2011) Plasma brain natriuretic peptide predicts death during hospitalization in acute ischaemic stroke and transient ischaemic attack patients with atrial fibrillation. Eur J Neurol 18: 165–169. 20528912 10.1111/j.1468-1331.2010.03101.x

[11] Montaner J, Garcia-Berrocoso T, Mendioroz M, Palacios M, Perea-Gainza M, et al (2012) Brain natriuretic peptide is associated with worsening and mortality in acute stroke patients but adds no prognostic value to clinical predictors of outcome. Cerebrovasc Dis 34: 240–245. 23018289 10.1159/000341858

[12] Shibazaki K, Kimura K, Okada Y, Iguchi Y, Uemura J, et al (2009) Plasma brain natriuretic peptide as an independent predictor of in-hospital mortality after acute ischemic stroke. Intern Med 48: 1601–1606. 19755761 10.2169/internalmedicine.48.2166

[13] Rost NS, Biffi A, Cloonan L, Chorba J, Kelly P, et al (2012) Brain natriuretic peptide predicts functional outcome in ischemic stroke. Stroke 43: 441–445. 22116811 10.1161/STROKEAHA.111.629212 PMC3265658

[14] Mortezabeigi HR, Taghizadeh A, Talebi M, Amini K, Goldust M (2013) ABCD2 score and BNP level in patients with TIA and cerebral stroke. Pak J Biol Sci 16: 1393–1397. 24511754 10.3923/pjbs.2013.1393.1397

[15] Garcia-Berrocoso T, Giralt D, Bustamante A, Etgen T, Jensen JK, et al (2013) B-type natriuretic peptides and mortality after stroke: a systematic review and meta-analysis. Neurology 81: 1976–1985. 24186915 10.1212/01.wnl.0000436937.32410.32 PMC3854833

[16] Reiter M, Twerenbold R, Reichlin T, Benz B, Haaf P, et al (2012) Early diagnosis of acute myocardial infarction in patients with pre-existing coronary artery disease using more sensitive cardiac troponin assays. Eur Heart J 33: 988–997. 22044927 10.1093/eurheartj/ehr376

[17] Christensen H, Johannesen HH, Christensen AF, Bendtzen K, Boysen G (2004) Serum cardiac troponin I in acute stroke is related to serum cortisol and TNF-alpha. Cerebrovasc Dis 18: 194–199. 15273434 10.1159/000079941

[18] Di Angelantonio E, Fiorelli M, Toni D, Sacchetti ML, Lorenzano S, et al (2005) Prognostic significance of admission levels of troponin I in patients with acute ischaemic stroke. J Neurol Neurosurg Psychiatry 76: 76–81. 15607999 10.1136/jnnp.2004.041491 PMC1739298

[19] Faiz KW, Thommessen B, Einvik G, Brekke PH, Omland T, et al (2014) Determinants of high sensitivity cardiac troponin T elevation in acute ischemic stroke. BMC Neurol 14: 96. 24885286 10.1186/1471-2377-14-96 PMC4107722

[20] Katan M, Fluri F, Morgenthaler NG, Schuetz P, Zweifel C, et al (2009) Copeptin: a novel, independent prognostic marker in patients with ischemic stroke. Ann Neurol 66: 799–808. 20035506 10.1002/ana.21783

[21] Hatano S (1976) Experience from a multicentre stroke register: a preliminary report. Bull World Health Organ 54: 541–553. 1088404 PMC2366492

[22] Brott T, Adams HP Jr, Olinger CP, Marler JR, Barsan WG, et al (1989) Measurements of acute cerebral infarction: a clinical examination scale. Stroke 20: 864–870. 2749846 10.1161/01.str.20.7.864

[23] Johnston SC, Rothwell PM, Nguyen-Huynh MN, Giles MF, Elkins JS, et al (2007) Validation and refinement of scores to predict very early stroke risk after transient ischaemic attack. Lancet 369: 283–292. 17258668 10.1016/S0140-6736(07)60150-0

[24] Adams HP Jr, Bendixen BH, Kappelle LJ, Biller J, Love BB, et al (1993) Classification of subtype of acute ischemic stroke. Definitions for use in a multicenter clinical trial. TOAST. Trial of Org 10172 in Acute Stroke Treatment. Stroke 24: 35–41. 7678184 10.1161/01.str.24.1.35

[25] Tang WH, Wu Y, Nicholls SJ, Brennan DM, Pepoy M, et al Subclinical myocardial necrosis and cardiovascular risk in stable patients undergoing elective cardiac evaluation. Arterioscler Thromb Vasc Biol 30: 634–640. 20032289 10.1161/ATVBAHA.109.201210 PMC3045838

[26] Mingels A, Jacobs L, Michielsen E, Swaanenburg J, Wodzig W, et al (2009) Reference population and marathon runner sera assessed by highly sensitive cardiac troponin T and commercial cardiac troponin T and I assays. Clin Chem 55: 101–108. 18988757 10.1373/clinchem.2008.106427

[27] Reichlin T, Hochholzer W, Bassetti S, Steuer S, Stelzig C, et al (2009) Early diagnosis of myocardial infarction with sensitive cardiac troponin assays. N Engl J Med 361: 858–867. 19710484 10.1056/NEJMoa0900428

[28] Szabo K, Kern R, Gass A, Hirsch J, Hennerici M (2001) Acute stroke patterns in patients with internal carotid artery disease: a diffusion-weighted magnetic resonance imaging study. Stroke 32: 1323–1329. 11387494 10.1161/01.str.32.6.1323

[29] DeLong ER, DeLong DM, Clarke-Pearson DL (1988) Comparing the areas under two or more correlated receiver operating characteristic curves: a nonparametric approach. Biometrics 44: 837–845. 3203132

[30] Pencina MJ, D'Agostino RB Sr, Steyerberg EW (2011) Extensions of net reclassification improvement calculations to measure usefulness of new biomarkers. Stat Med 30: 11–21. 21204120 10.1002/sim.4085 PMC3341973

[31] Konig IR, Ziegler A, Bluhmki E, Hacke W, Bath PM, et al (2008) Predicting long-term outcome after acute ischemic stroke: a simple index works in patients from controlled clinical trials. Stroke 39: 1821–1826. 18403738 10.1161/STROKEAHA.107.505867

[32] Jimenez Caballero PE, Lopez Espuela F, Portilla Cuenca JC, Ramirez Moreno JM, Pedrera Zamorano JD, et al (2013) Charlson comorbidity index in ischemic stroke and intracerebral hemorrhage as predictor of mortality and functional outcome after 6 months. J Stroke Cerebrovasc Dis 22: e214–218. 23352682 10.1016/j.jstrokecerebrovasdis.2012.11.014

[33] Codognotto M, Piccoli A, Zaninotto M, Mion M, Plebani M, et al (2007) Renal dysfunction is a confounder for plasma natriuretic peptides in detecting heart dysfunction in uremic and idiopathic dilated cardiomyopathies. Clin Chem 53: 2097–2104. 17934072 10.1373/clinchem.2007.089656

[34] Idris I, Hill R, Ross I, Sharma JC (2010) N-terminal probrain natriuretic peptide predicts 1-year mortality following acute stroke: possible evidence of occult cardiac dysfunction among patients with acute stroke. Age Ageing 39: 752–755. 20846963 10.1093/ageing/afq098

[35] Etgen T, Baum H, Sander K, Sander D (2005) Cardiac troponins and N-terminal pro-brain natriuretic peptide in acute ischemic stroke do not relate to clinical prognosis. Stroke 36: 270–275. 15604421 10.1161/01.STR.0000151364.19066.a1

[36] Makikallio AM, Makikallio TH, Korpelainen JT, Vuolteenaho O, Tapanainen JM, et al (2005) Natriuretic peptides and mortality after stroke. Stroke 36: 1016–1020. 15802631 10.1161/01.STR.0000162751.54349.ae

[37] Foerch C, Montaner J, Furie KL, Ning MM, Lo EH (2009) Invited article: searching for oracles? Blood biomarkers in acute stroke. Neurology 73: 393–399. 19652144 10.1212/WNL.0b013e3181b05ef9 PMC2725929

[38] Katan M, Fluri F, Schuetz P, Morgenthaler NG, Zweifel C, et al (2010) Midregional pro-atrial natriuretic peptide and outcome in patients with acute ischemic stroke. J Am Coll Cardiol 56: 1045–1053. 20846604 10.1016/j.jacc.2010.02.071

[39] Jensen JK, Kristensen SR, Bak S, Atar D, Hoilund-Carlsen PF, et al (2007) Frequency and significance of troponin T elevation in acute ischemic stroke. Am J Cardiol 99: 108–112. 17196472 10.1016/j.amjcard.2006.07.071

[40] Jensen JK, Ueland T, Aukrust P, Antonsen L, Kristensen SR, et al (2012) Highly sensitive troponin T in patients with acute ischemic stroke. Eur Neurol 68: 287–293. 23051820 10.1159/000341340

[41] Rizos T, Guntner J, Jenetzky E, Marquardt L, Reichardt C, et al (2012) Continuous stroke unit electrocardiographic monitoring versus 24-hour Holter electrocardiography for detection of paroxysmal atrial fibrillation after stroke. Stroke 43: 2689–2694. 22871678 10.1161/STROKEAHA.112.654954

[42] Hendrikx T, Hornsten R, Rosenqvist M, Sandstrom H (2013) Screening for atrial fibrillation with baseline and intermittent ECG recording in an out-of-hospital population. BMC Cardiovasc Disord 13: 41. 23758799 10.1186/1471-2261-13-41 PMC3682914

