# Supplementary material for: BNP but Not s-cTnln Is Associated with Cardioembolic Aetiology and Predicts Short and Long Term Prognosis after Cerebrovascular Events
Source: PLoS One. 2014 Jul 29;9(7):e102704. doi: 10.1371/journal.pone.0102704 (PMC4114527; doi:10.1371/journal.pone.0102704)
Supplement: Protocol S1 — Trial protocol. (DOC) [file pone.0102704.s002.doc]

Copeptin as a novel diagnostic and prognostic marker in the management of neurological and neurosurgical patients with sodium imbalance

The „COSMOS“-Study

(Copeptin in Osmoregulation and Stress assessment)

Mira Katan, , Christian Zweifel, Nils Morgenthaler, Philipp Schütz, Stephan Engelter, Michael Woydt, Jonas Rutishauser, Christian Müller, Roland Bingisser , Andreas Steck, Beat Müller and Mirjam Christ-Crain

**Correspondence:**

Dr. Mira Katan, Department of Neurology, University Hospital Basel , CH-4031 Basel, Switzerland

Email: katanm@uhbs.ch; T: +41 61 265 2525; F: +41 61 265 4800; H: +41 79 208 90 66

#### 1. SYNOPSIS

**Background:**

Sodium imbalance is common and an adverse prognostic factor in hospitalized patients. However, identifying the causes of sodium imbalance is challenging in clinical practice. Levels of antidiuretic hormone (ADH) are elevated in patients with stroke correlating with disease severity and stress level; however, its measurement is cumbersome. ADH is derived from a larger precursor peptide along with Copeptin, which is a more stable peptide directly mirroring the production of ADH. Copeptin can be assayed readily in plasma. Early prognostic factors to predict in-hospital mortality and medium/long-term outcome in critically ill neurological patients, are helpful to guide and tailor early decisions on treatment, discharge from the intensive care unit and application of interventions to prevent deterioration of neurological functions.

**Aim:** To evaluate Copeptin as a diagnostic tool in disturbances of water homeostasis and prognostic tool to predict outcome in a well-defined cohort of stroke patients and patients undergoing intracranial surgery.

**Design:** Prospective, observational study.

**Location Setting:** Emergency and neurogical and neurosurgical clinic of the University Hospital of Basel.

**Patients:** Neurological patients with ischemic and hemorrhagic stroke and patients undergoing intracranial surgery.

**Intervention:** After informed consent, all routinely determined baseline data will be assessed including medical history, clinical items (i.e. neurological status, volume status, pulse rate, blood pressure, weight) and laboratory items (i.e. urine / serum osmolality, electrolytes, among others). All patients will have a follow-up with clinical and laboratory assessment until the day of discharge. After 3 months, they will be followed-up by a structured telephone interview to assess outcome (mortality, morbidity, as assessed by the ranking scale and Barthel index). Copeptin will be assessed in a batch analysis upon completion of the plasma asseveration.

**Variables and measurements:** Baseline data on medical history and clinical items, co-morbidities and treatment procedures will be collected. This exploratory study will be conducted over a 12 month period from November 2006 to November 2007.

**Study hypothesis:** 1. Copeptin will improve the diagnostic accuracy to diagnose sodium imbalances as compared to routinely used markers 2. Copeptin will be a reliable prognostic tool, dependent or independent of sodium imbalance, to predict short-term (i.e. in-hospital) and medium-term (i.e. 3 months) clinical outcome in stroke patients. Further other biomarkers will be measured to assess prognostic values.

**Analysis:** Our sample size consideration is based on the second prognostic question to predict the outcome of neurological and neurosurgical patients. To determine an optimal clinical model we will undertake a multivariable regression analysis to assess which variables are independently associated with outcome. Multiple regression models with a minimum of 10 to 15 observations per predictor variable reveal good estimates. We will evaluate 18 predictors in our multivariate analysis. Therefore, we aim to include a minimum sample size of 180 patients (for 10 observations per predictor) to 270 (for 15 observations per predictor) and a maximum sample size of 360 patients (for 20 observations per predictor).

**Significance:** Despite the frequent occurrence and the poor outcomes of serious disorders of sodium balance, few controlled data are available to guide the clinician. A better diagnostic approach to determine the etiology of hyponatremia should improve patient management. Copeptin as a prognostic marker in neurological and neurosurgical patients could become an innovative tool to guide early treatment decisions, discharge from the stroke unit and application of interventions.

**2. RESEARCH PLAN**

**2.1 Introduction**

Disorders of serum sodium are the most common electrolyte disorder in hospital practice [1] and represent a significant healthcare burden [2]. Mild hyponatremia (i.e. plasma sodium 130-135mmol/l) is found in as many as 15-30% of hospitalized patients [3-6]. More severe hyponatremia (plasma sodium <130mmol/l) is seen in 1-4 % of inpatients [7]. Hyponatremia in hospitalized patients has a significant mortality ranging from 5% to 50%, depending on severity and acuity of onset as well as comorbidities. Hypernatremia is far less common but mortality ranges from 60 to 75% [8].

The central nervous system (CNS) plays an integral role in the neuroendocrine regulation of sodium and water homeostasis. Hyponatremia is the most common electrolyte disorder encountered in the neurologic intensive care unit [3,9,10]. It is seen in patients with subarachnoidal hemorrhage (SAH) [11], brain tumors [12-14] and ischemic stroke [15-17], complicating their clinical courses [9]. A timely and accurate diagnosis followed by a correct treatment is crucial [18,19]. Differential diagnoses include the syndrome of inappropriate antidiuretic hormone secretion (SIADH), cerebral salt wasting (CSW), hypo- or hypervolemic hyponatremia, and mixed cases [20]. Besides measurement of plasma osmolality and sodium concentration in urine, determination of the extracellular fluid volume remains the most important discriminatory element. However, its prediction based on clinical signs and routine laboratory evaluation has a limited sensitivity and specificity of <50% [21,22]. Accordingly, only 10% of physicians reach the correct diagnosis of the underlying cause of hyponatremia with the routinely used clinical diagnostic algorithms [23]. Thus, there is demand for more reliable parameters to improve the diagnosis of sodium imbalance in patients with CNS disease.

Plasma sodium concentrations are kept within a narrow range to maintain a constant plasma osmolality. This is predominantly regulated by arginin vasopressin (ADH). ADH is produced in the hypothalamus and stored in and released from the neurohypophysis. Plasma ADH levels begin to fall immediately upon swallowing water, before plasma dilution occurs, allowing prompt excretion of diluted urine [24]. Unfortunately, the measurement of circulating ADH levels is challenging, since the mature hormone is very unstable, largely attached to platelets and rapidly cleared from plasma with a half-life of 5 to 15 minutes. ADH is derived from a larger precursor peptide (pre-provasopressin) along with two other peptides, neurophysin II and copeptin. Released in an equimolar ratio, the amount of copeptin mirrors the production of ADH. Plasma copeptin concentration have recently been shown to be an easy to determine, steady parameter [25,26].

ADH plays not only a critical role in osmo-regulation, but also in the regulation of the hypothalamo-pituitary-adrenal (HPA) axis and, thus, reflecting the individual stress response. “Stressors” such as stroke or hemorrhage are strong stimulators of the release of ADH [27]. Accordingly, elevated ADH levels have been reported in experimental ischemia [28,29]. In a small clinical study, patients with stroke had increased ADH levels as compared to controls, correlating with stroke severity [30]. Early prognostic factors to predict in-hospital mortality and medium/long-term outcome in stroke patients are important to guide and tailor early decision on treatment, discharge from the stroke unit and interventions to prevent deterioration of mobility status. Existing prognostic models for stroke often include impractical predictor variables, lack sufficient predictive accuracy and are, thus, not routinely used. In this context, copeptin may be a new prognostic marker and a helpful tool in the early risk stratification of stroke patients.

**2.2 DETAILED RESEARCH PLAN**

**A. Objectives**

The objectives of this trial are to evaluate

1. The diagnostic value of copeptin levels in the diagnosis of water imbalance in patients with stroke and patients undergoing intracranial surgery.
2. The prognostic accuracy of copeptin levels and other biomarkers to predict the clinical outcome in patients with stroke and patients undergoing intracranial surgery.

Specific objectives of the study are:

- - 1. **Diagnostic Study:**

**Hyponatremia** will be defined as serum sodium concentration below 135 mmol/L [31].

Specifically, we aim to investigate whether Copeptin levels can facilitate the differential diagnosis of hyponatraemia. Thereby, we will use a standardized clinical algorithm based on medical history, drug history, clinical assessment of the extracellular fluid volume, and laboratory parameters such as plasma and urine sodium concentration, plasma and urine osmolality, creatinine, uric acid, urea, total protein, albumin, TSH, fT4, T3, blood glucose, urea nitrogen, plasma potassium to evaluate the aetiology of hyponatraemia (**figure 1**). In our cohort, wee expect a prevalence of around 20 % (i.e. around 75 patients of the 350 patients derived from the power calculation, see below) to have hyponatraemia. The etiologies are listed in Figure 1 including the estimated relative contributions and the most frequent diagnosis within the subgroup is underlined.

**Figure 1** Diagnostic algorithm of hypotonic hyponatraemia:

We **hypothesize** that overall, included patients will have moderately but significantly higher copeptin levels (around 5pmol/L) as compared to healthy controls (4pmol/L). In patients with hyponatraemia, copeptin levels will be lower as compared to patients without hyponatraemia, however, with a wide range (1.7pmol/L (detection limit) to 15pmol/L). Analysing the subgroup of patients with hyponatraemia, we assume that in patients with SIADH, copeptin levels will be markedly increased (i.e. 12pmol/L), moderately increased (8pmol/L) in hypovolemic hyponatraemia and suppressed in hypervolemic hyponatraemia (<2pmol/L). Importantly, we anticipate to identify SIADH as a co-factor also in some patients with “typical” hypervolaemic and hypovolemic hyponatremia, where ADH disturbances have been neglected previously due to the incapability to assess circulating ADH levels.

**Hypernatremia**

Hypernatremia will be defined as a rise in the serum sodium concentration to a value exceeding 145 mmol/L [3]. Again, we will use a standardized clinical algorithm based on clinical and laboratory parameters to evaluate the aetiology of hypernatraemia.

Of all 350 patients included (see sample size considerations) we expect 10 % (i.e. around 45 patients) to have hypernatraemia. The etiologies are listed in Figure 1 including the estimated relative contributions and the most frequent diagnosis within the subgroup underlined in red..

**Figure 2** Diagnostic algorithm of hypernatraemia

We **hypothesize** that in patients with hypernatraemia, copeptin levels overall will be higher as compared to patients without hyponatraemia, however, with a wide range (1.7pmol/L (detection limit) to 12pmol/L). Analysing the subgroup of patients with hypernatraemia, we assume that in patients with Diabetes insipidus, copeptin levels will be markedly decreased (i.e. 1.7-2pmol/L), moderately increased (8pmol/L) in hypervolemic hyponatraemia and markedly increased in hypervolemic hyponatraemia (12pmol/L).

**Statistical Analyses**

Copeptin levels will be assessed in a blinded batch analysis upon completion of the plasma asseveration.

Discrete variables will be expressed as counts (percentage) and continuous variables as means ± standard deviation (SD) or median (interquartile range), unless stated otherwise. First, the overall prevalence of hyponatraemia in neurological and neurosurgical patients will be assessed. Frequency comparison will be done by chi-square test. Two-group comparison of normally distributed data will be performed by Students t-test. For multigroup comparisons, one-way analysis of variance with least square difference for posthoc comparison will be applied. For data not normally distributed, the Mann-Whitney-U test is used if only two groups are compared. The Kruskal-Wallis one-way analysis of variance with Bonferroni’s multiple posthoc test will be used if more than two groups were being compared.

Furthermore we will do a correlation analysis of copeptin levels with sodium levels, serum osmolality and other diagnostic laboratory parameters. Correlation analyses will be performed by using Spearman rank correlation. Levels that are non-detectable will be assigned a value equal to the lower limit of detection for the assay. All testing will be two-tailed and P values less than 0.05 were considered to indicate statistical significance. Scatterplot data will be shown with GraphPad Prism®, Version 4.00 for Windows (GraphPad Software, San Diego California, USA).

We will then compare the diagnostic accuracy of Copeptin levels with other potential biomarkers for sodium imbalance, e.g. atrial natriuretic peptide (ANP) and B-type natriuretic peptide (BNP). The ability of Copeptin to foresee sodium disbalance before it becomes evident by laboratory analysis will be assessed. Therefore we will correlate Copeptin levels with sodium level changes over time.We will construct receiver operating characteristic (ROC) curves and determine the areas under the receiver operating characteristic curve (AUC). The sensitivity and specificity to predict the correct diagnosis of hyponatraemia will be determined, based on the above standardized algorithm [32]. This ascertainment will be done retrospectively on the basis of the review of the complete patient charts by two study investigators, blinded to each other’s diagnosis. Discordant cases will be mutually discussed and resolved. Patients will be managed as usual according to the state of the art treatment.

**2.2.2. Prognostic Study:**

We will assess the prognostic value of copeptin dependent or independent of sodium imbalance.

**Prognostic question in patients with stroke**

In stroke patients we will evaluate the prognostic value of Copeptin -levels - dependent or independent of sodium imbalance- to predict mortality, ICU stay, length of hospitalization; and morbidity (i.e. outcome after 90 days), as assessed by the National Institute of Health and Stroke Scale (NIHSS, [33], **Appendix 1**) on admission and at day 5, the Barthel index [34] (**Appendix 2**) and Ranking scale [35] (**Appendix 3**) at day 5 of the hospitalization and after a follow-up of 3 months by telephone interview with the patient or if not possible with the nearest relative or health care provider. Further other biomarkers, such as MR-pro ANP, BNP and Troponin will be assessed.

**Prognostic question in neurosurgical patients**

In neurosurgical patients, we will evaluate the prognostic accuracy of Copeptin levels to predict mortality, prolonged ICU stay, length of hospitalization, and morbidity (i.e. outcome after 90 days), as assessed by the Glasgow coma scale **(Appendix 4)** on admission and at day 5**,** Glasgow outcome Scale (**Appendix 5**) and Barthel index at day 5 of the hospitalization and after a follow-up of 3 months by a telephone interview with the patient or if not possible with the nearest relative or health care provider.

**2.3.** We will assess other potential prognostic factors (i.e. pro-atrial natriuretic peptide, B-type natriuretic peptide, procalcitonin, C-reactive protein, IL-6, neuron specific enolase, Cu/Zn superoxide dismutase (Cu/Zn SOD) [36]).

**Overall, the hypotheses are the following**:

1. Copeptin will be a new accurate diagnostic tool to facilitate the differential diagnosis of the etiology of sodium imbalance. Copeptin will have a higher diagnostic accuracy as compared to the routinely used clinical assessment of the extracellular fluid volume by physical examination and laboratory parameters. If copeptin is found to be an accurate tool, we aim to validate our findings in a future intervention study, with the aim to improve the management of neurosurgical and neurological patients with sodium imbalance based on copeptin levels.

2. Copeptin will be a reliable prognostic factor and high-quality predictor for the clinical outcome (i.e. mortality, length of stay in ICU, discharge from ICU, length of hospitalization, clinical outcome within the first week and after three months) of neurological and neurosurgical patients.

**Statistical Analyses**

Basic statistical analyses will be performed as described in the diagnostic study. We will evaluate 18 known predictors (i.e. *age; sex; clinical features* (NIHSS or GCS), *important comorbidities* (coronary artery disease, heart failure, atrial fibrillation, hypertension on admission, previous TIA/Stroke, renal disease, dementia, diabetes); *location of the lesion* (LACS etc.); *stroke subtype by the TOAST criteria*; *therapies* (thrombolysis); *laboratory parameters (*baseline glucose level on admission [46-50], CRP); *body temperature*; *smoking histo*ry), and copeptin levels in our multivariate analysis. All variables will first be tested one by one against the dependent variable unfavorable outcome (defined either as Barthel index <85 or modified Ranking scale 3 to 6) for the presence of a significant association. Only variables significantly associated with the outcome will be assessed in a multivariate model performed separately for each parameter.

**B. Study Design**

**Study setting**

Emergency and neurogical and neurosurgical clinic of the University Hospital of Basel

**Inclusion criteria**

1. All consecutive patients who are admitted to the emergency department with an ischemic or hemorrhagic stroke or transient ischemic attach (TIA) according to the World Health organization criteria [37] with symptom onset within the last 3 days.

2. All consecutive patients who undergo intracranial surgery due to

- pituitary tumors

- intracerebral hemorrhage (ICH)

- subarachnoidal hemorrhage (SAH)

- chronic subdural hematoma

- head trauma with contusio cerebri

- intracranial abcesses

**Exclusion criteria**

Patients without informed consent.

**C Procedures**

**Baseline data collection (Appendix 6)**

Access to data of all eligible patients that are not included into this study is important to avoid a selection bias. Thus, we will collect baseline data and information on inclusion and exclusion criteria in all eligible patients irrespective whether they are or are not included into the study. This will allow the comparison of baseline data of eligible patients who consented to participate with those who did not.

Baseline data collection in patients will be collected by the investigators and contain

a) age

b) gender

c) BMI

d) Medical history items: actual history that preceded the hospitalization; ABCD score in patients with transient ischemic attack (Rothwell [38]**Appendix 7**); family history; relevant co-morbidities also assessed by the charlson index [39] (**Appendix 8**) (i.e. hypertension, previous stroke, previous TIA, ischemic heart disease, atrial fibrillation, diabetes mellitus, renal and liver dysfunction, congestive heart failure, dyslipidemia; comorbidities with the risk of hyponatremia (severe hypothyroidism, glucocorticoid insufficiency, neoplasm, HIV infection); smoking history (pack-years) and status (pack per day); current medication; alcohol consumption (glass and grams per day); time from onset of symptoms to admission.

e) Place of residence: i.e. independent living, defined as living at home or in an old people’s home with or without support of the family circle and/or professional care (the family circle consists of the spouse and/or other important persons who live together with the patient; dependent living, defined as nursing home long-stay department, other hospital [40].

f) Clinical items: physical examination including neurological status, NIHSS (to assess the severity of stroke in stroke patients) and Glasgow Coma scale (GCS [41]); blood pressure, pulse rate, weight, volume status (including skin turgor, jugular venous distension, auscultation, if available flow sheet of fluid intake and loss), body temperature; in neurosurgical patients intracerebral pressure if performed within the routine clinical management.
g) Clinical symptoms of hyponatraemia will be evaluated on admission and in case of sodium imbalance in neurological patients. In patients undergoing intracranial surgery we will evaluate clinical symptoms daily. Specifically we will monitor the presence of headache, anorexia, nausea, vomiting, muscle cramps and aches, seizures, confusion, apathetic or lethargic development.
h) Routine/Standard laboratory tests: routine blood sampling including: hematocrit, blood urea nitrogen, bicarbonate, total protein, albumin, uric acid serum and urine electrolytes, urine and serum osmolality, creatinine, lipids, TSH, fT4, T3, and basal cortisol. All blood sampling will be done before any food intake, or smoking, if feasible (both, upright position and smoking will potentially elevate ADH and Copeptin levels). Alternatively, influencing factors will be monitored.
i) Imaging : Computer tomography or MRI of the neurocranium (T1, T2, diffusion-weighted image sequence, with or without contrast), if indicated magnetic resonance angiography or conventional cerebral angiography. We will record the time-points of contrast agent application.

Stroke patients will also be classified on the basis of the vascular territory of the ischemic lesion as follows: total anterior circulation syndrome (TACS), partial anterior circulation syndrome (PACS), lacunar circulation syndrome (LACS), posterior circulation syndrome (POCS).

j) Further investigations: Stroke patients will have neurosonography, echocardiography, standard 12-leaf electrocardiography and 24 –hour electrocardiography and then will be classified by etiology of strokes according to Trial of Org 10172 in acute Treatment (TOAST) stroke subtype classification, which differs between large artery atherosclerosis, cardioembolism, small-artery occlusion, other etiology, and undetermined etiology [42].

Importantly, all these investigations are currently performed in the routine setting. Therefore, no additional costs will be charged to the patient, the health care provider or the health insurances.

**Informed consent statement (Appendix 9 a and b,10,11,12)**

The study will be approved by the ethics committee of Basel (Ethikkommission beider Basel). It is important to note that this is an exploratory and observational study; the only study related intervention will be the asseveration of 7.5ml of plasma obtained during the routinely performed blood sampling. Therefore, patients are required to provide written informed consent that they agree for the use of their data for scientific purposes. In patients, in which “informed consent” is not feasible due to sequela of the acute CNS lesion (the latter a prerequisite for inclusion), patients’ next to kin can sign an assent form to state the presumptive will of the patient. In case, next of kin are not readily available, a treating physician – who must not be involved in the study – have to certify that there are no objections for inclusion in the study from his point of view. Only after these informed consent procedures the patient can be included in the study.

**Management of participants throughout the trial**

Step 1. All eligible patients in the emergency department or the neurological ward or the neurosurgical ward will be included into the study.

Step 2. All baseline data will be collected.

Step 3. During hospitalization we Clinical items including weight, blood pressure, pulse rate, volume status and body temperature will be assessed by chart review until discharge. In neurosurgical patients, we will monitor intracerebral pressure if performed within the routine clinical management.

- Fluid treatment and drugs

- Potential symptoms of hyponatremia, i.e. headache, nausea, vomiting, muscle cramps and aches, anorexia, impaired consciousness, seizure **(Appendix13)** [9].

- Routinely performed laboratory tests (chemogramm, plasma glucose, serum osmolality, urine osmolality, sodium in urine, hematocrit) will be sampled at the time-points when blood sampling is routinely done on the wards.

Step 4. In all patients, on day 5 of the hospitalization, a clinical examination with GCS, NIHSS, Barthel Index and Ranking Scale will be performed.

The future place of residence (i.e. dependent vs. independent living) will be assessed.

Step 6. In patients with ischemic stroke a telephone follow-up **(Appendix 14)** regarding morbidity and mortality (as assessed by the Barthel Index and Ranking Scale) will be obtained after 3 months. An unfavorable outcome will be defined as a Barthel index <85 or modified Rankin scale of 3 to 6 [43].

Patients after neurosurgery will have a telephone follow-up **( Appendix 15)** after 3 months to evaluate mortality, relapse, and quality of life as assessed by the Glasgow outcome scale [44] and Barthel index.

**Measurement of copeptin concentrations**

Copeptin levels will be measured with a new chemiluminescens sandwich immunoassay with a lower detection limit of the assay of 0.4 pmol/L. Therefore, we will sample an additional 7.5ml Plasma EDTA tube which is asserved during the respective routine blood samplings. No additional blood sampling will be done.

**D. Potential risks, Adverse events and Monitoring board**

We consider the risks of this study to be minor and limited to the following

Blood Loss: 7.5ml of EDTA-plasma will be additionally drawn during each routine blood sampling. The total additional blood loss for this study will be less than 50 ml.

**E. Sample Size considerations**

Design

This is a prospective observational study to evaluate Copeptin as a diagnostic and prognostic marker in neurological and neurosurgical patients.

Analysis

Analysis tools

All relevant clinical and laboratory parameters obtained by interview, clinical tests and reviewing of the medical records will be entered into an Excel® database. Statistical Analysis System (SAS® Institute, Cary, NC, USA) and R for Windows (R Foundation for Statistical Computing, www.r-project.org) will be used for data analysis. Statistical evaluation will include stepwise logistic regression analysis to assess which variables are independently associated with outcome.

Sample size considerations

Our sample size consideration is based on our intention to determine an optimal clinical model to predict the outcome of neurological and neurosurgical patients (based on Barthel index, Rankin scale NIHSS scale and Glasgow outcome scale) (i.e. the prognostic question of the study). To determine an optimal clinical model we will undertake a multivariable regression analysis. It has been demonstrated that multiple regression models with a minimum of 10 to 15 observations per predictor variable reveal good estimates [45]. We will evaluate 18 known predictors (i.e. *age; sex; clinical features* (NIHSS or GCS), *important comorbidities* (coronary artery disease, heart failure, atrial fibrillation, hypertension on admission, previous TIA/Stroke, renal disease, dementia, diabetes); *location of the lesion* (LACS etc.); *stroke subtype by the TOAST criteria*; *therapies* (thrombolysis); *laboratory parameters (*baseline glucose level on admission [46-50], CRP); *body temperature*; *smoking histo*ry) in our multivariate analysis. Therefore, we aim to include a minimum sample size of 180 patients (for 10 observations per predictor) to 270 (for 15 observations per predictor) and a maximum sample size of 360 patients (for 20 observations per predictor). All variables will first be tested one by one against the dependent variable unfavorable outcome (defined either as Barthel index <85 or modified Ranking scale 3 to 6) for the presence of a significant association. Only variables significantly associated with the outcome will be assessed in a multivariate model performed separately for each parameter.

Based on the last year’s data in our hospital, the following number of patients with stroke and neurosurgery will be eligible:

*Patients*

|  | Cases 2003 | Cases 2004 |
| --- | --- | --- |
| Estimated eligible neurosurgical patients | Around 180 | Around 180 |
| Ischemic stroke | Around 300 | Around 300 |

An estimated total of around 480 patients will thus be eligible. With an estimated informed consent of 80% and an estimated loss of follow-up of 10%, approximately 350 patients could be included.

**F. Limitations**

**Confidentiality**

The names of the patients (for the purpose of the interviews) will be known only to the data centre. This information will be kept strictly confidential. All data forms will be handled as confidential information.

**G. Regulatory and liability considerations**

**Regulatory Considerations**

This study will be conducted in accordance with the ethical principles stated in the most recent version of the Declaration of Helsinki or the applicable International Conference on Harmonization (ICH) guidelines on good clinical practice, whichever represents the greater protection of the individual.

The study will be submitted to the institutional review board and registered in the Current Controlled Trials Database as “COSMOS” study. Written informed consent will be obtained from all included patients or their legal representatives. The study will not be started until approval of the study protocol by the local ethical committee. Data collected will be kept confidential and accessible only to researcher involved and the ethical committee.

**Liability**

This is an investigator driven study. The study procedures will be covered by the general liability insurance of the University Hospital Basel. Liability for the technical reliability of the Copeptin measurement is with the producer of the test (Brahms, Hennigsdorf, Germany). There are no additional costs for the patient or the health insurance to bear.

**2.3 TIMETABLE**

Start patient enrolment October 1^st^ 2006

End of patient recruitment October 1^st^ 2007

End of patient follow-up February 1^st^ 2008

Data entering & checking completed February 28^th^ 2008

Start of data analysis March 1^st^ 2008

End of data analysis April 30^st^ 2008

First draft of manuscript June 30^st^ 2008

**2.4 Significance of the proposed study**

Sodium imbalance is common and can be a life-threatening problem. Therefore a correct and early diagnosis is important. Early prognostic factors to predict in-hospital mortality and medium/long-term outcome in stroke patients and patients which undergo intracerebral surgery are crucial to guide and tailor early treatment decisions, discharge from the stroke unit and application of interventions to prevent deterioration of mobility status. Copeptin may be an important new diagnostic and prognostic marker in the management of patients with disruption of the neuroendocrine homeostasis.

Little evidence from randomized controlled trials exists for the treatment of sodium disorders.

Dependent on the results, we will in a next step perform intervention studies to evaluate whether Copeptin levels provide a useful tool to guide fluid management in these patients and to guide prognostic decisions in stroke patients.

**2.5. References**

1. Verbalis JG: Disorders of body water homeostasis. Best Pract Res Clin Endocrinol Metab 2003;17:471-503.

2. Boscoe A, Paramore C, Verbalis JG: Cost of illness of hyponatremia in the United States. Cost Eff Resour Alloc 2006;4:10.

3. Adrogue HJ, Madias NE: Hypernatremia. N Engl J Med 2000;342:1493-1499.

4. Ishikawa SE, Schrier RW: Pathophysiological roles of arginine vasopressin and aquaporin-2 in impaired water excretion. Clin Endocrinol (Oxf) 2003;58:1-17.

5. Smith DM, McKenna K, Thompson CJ: Hyponatraemia. Clin Endocrinol (Oxf) 2000;52:667-678.

6. Miller M, Morley JE, Rubenstein LZ: Hyponatremia in a nursing home population. J Am Geriatr Soc 1995;43:1410-1413.

7. Reynolds RM, Seckl JR: Hyponatraemia for the clinical endocrinologist. Clin Endocrinol (Oxf) 2005;63:366-374.

8. Kumar S, Berl T: Sodium. Lancet 1998;352:220-228.

9. Rabinstein AA, Wijdicks EF: Hyponatremia in critically ill neurological patients. Neurologist 2003;9:290-300.

10. Cole CD, Gottfried ON, Liu JK, Couldwell WT: Hyponatremia in the neurosurgical patient: diagnosis and management. Neurosurg Focus 2004;16:E9.

11. Palmer BF: Hyponatremia in patients with central nervous system disease: SIADH versus CSW. Trends Endocrinol Metab 2003;14:182-187.

12. Atkin SL, Coady AM, White MC, Mathew B: Hyponatraemia secondary to cerebral salt wasting syndrome following routine pituitary surgery. Eur J Endocrinol 1996;135:245-247.

13. Boehnert M, Hensen J, Henig A, Fahlbusch R, Gross P, Buchfelder M: Severe hyponatremia after transsphenoidal surgery for pituitary adenomas. Kidney Int Suppl 1998;64:S12-14.

14. Hensen J, Henig A, Fahlbusch R, Meyer M, Boehnert M, Buchfelder M: Prevalence, predictors and patterns of postoperative polyuria and hyponatraemia in the immediate course after transsphenoidal surgery for pituitary adenomas. Clin Endocrinol (Oxf) 1999;50:431-439.

15. Wijdicks EF, Vermeulen M, Hijdra A, van Gijn J: Hyponatremia and cerebral infarction in patients with ruptured intracranial aneurysms: is fluid restriction harmful? Ann Neurol 1985;17:137-140.

16. Kamoi K, Toyama M, Takagi M, Koizumi T, Niishiyama K, Takahashi K, Sasaki H, Muto T: Osmoregulation of vasopressin secretion in patients with the syndrome of inappropriate antidiuresis associated with central nervous system disorders. Endocr J 1999;46:269-277.

17. Goldberg M, Handler JS: Hyponatremia and renal wasting of sodium in patients with malfunction of the central nervous system. N Engl J Med 1960;263:1037-1043.

18. Anderson RJ: Hospital-associated hyponatremia. Kidney Int 1986;29:1237-1247.

19. Berl T: Treating hyponatremia: damned if we do and damned if we don't. Kidney Int 1990;37:1006-1018.

20. Sherlock M, O'Sullivan E, Agha A, Behan LA, Rawluk D, Brennan P, Tormey W, Thompson CJ: The incidence and pathophysiology of hyponatraemia after subarachnoid haemorrhage. Clin Endocrinol (Oxf) 2006;64:250-254.

21. Musch W, Thimpont J, Vandervelde D, Verhaeverbeke I, Berghmans T, Decaux G: Combined fractional excretion of sodium and urea better predicts response to saline in hyponatremia than do usual clinical and biochemical parameters. Am J Med 1995;99:348-355.

22. Chung HM, Kluge R, Schrier RW, Anderson RJ: Clinical assessment of extracellular fluid volume in hyponatremia. Am J Med 1987;83:905-908.

23. Hoorn EJ, Halperin ML, Zietse R: Diagnostic approach to a patient with hyponatraemia: traditional versus physiology-based options. Qjm 2005;98:529-540.

24. Seckl JR, Williams TD, Lightman SL: Oral hypertonic saline causes transient fall of vasopressin in humans. Am J Physiol 1986;251:R214-217.

25. Struck J, Morgenthaler NG, Bergmann A: Copeptin, a stable peptide derived from the vasopressin precursor, is elevated in serum of sepsis patients. Peptides 2005;26:2500-2504.

26. Morgenthaler NG, Struck J, Alonso C, Bergmann A: Assay for the Measurement of Copeptin, a Stable Peptide Derived from the Precursor of Vasopressin. Clin Chem 2005.

27. Itoi K, Jiang YQ, Iwasaki Y, Watson SJ: Regulatory mechanisms of corticotropin-releasing hormone and vasopressin gene expression in the hypothalamus. J Neuroendocrinol 2004;16:348-355.

28. Chang Y, Chen TY, Chen CH, Crain BJ, Toung TJ, Bhardwaj A: Plasma Arginine-Vasopressin Following Experimental Stroke: Effect of Osmotherapy. J Appl Physiol 2005.

29. Joynt RJ, Feibel JH, Sladek CM: Antidiuretic hormone levels in stroke patients. Ann Neurol 1981;9:182-184.

30. Barreca T, Gandolfo C, Corsini G, Del Sette M, Cataldi A, Rolandi E, Franceschini R: Evaluation of the secretory pattern of plasma arginine vasopressin in stroke patients. Cerebrovasc Dis 2001;11:113-118.

31. Adrogue HJ, Madias NE: Hyponatremia. N Engl J Med 2000;342:1581-1589.

32. Reynolds RM, Padfield PL, Seckl JR: Disorders of sodium balance. Bmj 2006;332:702-705.

33. Brott T, Adams HP, Jr., Olinger CP, Marler JR, Barsan WG, Biller J, Spilker J, Holleran R, Eberle R, Hertzberg V, et al.: Measurements of acute cerebral infarction: a clinical examination scale. Stroke 1989;20:864-870.

34. Collin C, Wade DT, Davies S, Horne V: The Barthel ADL Index: a reliability study. Int Disabil Stud 1988;10:61-63.

35. de Haan R, Limburg M, Bossuyt P, van der Meulen J, Aaronson N: The clinical meaning of Rankin 'handicap' grades after stroke. Stroke 1995;26:2027-2030.

36. Lees KR, Zivin JA, Ashwood T, Davalos A, Davis SM, Diener HC, Grotta J, Lyden P, Shuaib A, Hardemark HG, Wasiewski WW: NXY-059 for acute ischemic stroke. N Engl J Med 2006;354:588-600.

37. Organisation WH: Proposal for the multinational monitoring of trends and determinants in cardiovascular disease( MONICA project). WHO/MNC/82.1 Rev 1. 1983.

38. Rothwell PM, Giles MF, Flossmann E, Lovelock CE, Redgrave JN, Warlow CP, Mehta Z: A simple score (ABCD) to identify individuals at high early risk of stroke after transient ischaemic attack. Lancet 2005;366:29-36.

39. Goldstein LB, Samsa GP, Matchar DB, Horner RD: Charlson Index comorbidity adjustment for ischemic stroke outcome studies. Stroke 2004;35:1941-1945.

40. Meijer R, van Limbeek J, Peusens G, Rulkens M, Dankoor K, Vermeulen M, de Haan RJ: The Stroke Unit Discharge Guideline, a prognostic framework for the discharge outcome from the hospital stroke unit. A prospective cohort study. Clin Rehabil 2005;19:770-778.

41. Teasdale G, Jennett B: Assessment of coma and impaired consciousness. A practical scale. Lancet 1974;2:81-84.

42. Adams HP, Jr., Bendixen BH, Kappelle LJ, Biller J, Love BB, Gordon DL, Marsh EE, 3rd: Classification of subtype of acute ischemic stroke. Definitions for use in a multicenter clinical trial. TOAST. Trial of Org 10172 in Acute Stroke Treatment. Stroke 1993;24:35-41.

43. Etgen T, Baum H, Sander K, Sander D: Cardiac troponins and N-terminal pro-brain natriuretic peptide in acute ischemic stroke do not relate to clinical prognosis. Stroke 2005;36:270-275.

44. Jennett B, Bond M: Assessment of outcome after severe brain damage. Lancet 1975;1:480-484.

45. Babyak MA: What you see may not be what you get: a brief, nontechnical introduction to overfitting in regression-type models. Psychosom Med 2004;66:411-421.

46. Witt BJ, Ballman KV, Brown RD, Jr., Meverden RA, Jacobsen SJ, Roger VL: The incidence of stroke after myocardial infarction: a meta-analysis. Am J Med 2006;119:354 e351-359.

47. Fischer U, Arnold M, Nedeltchev K, Schoenenberger RA, Kappeler L, Hollinger P, Schroth G, Ballinari P, Mattle HP: Impact of comorbidity on ischemic stroke outcome. Acta Neurol Scand 2006;113:108-113.

48. Hankey GJ: Long-term outcome after ischaemic stroke/transient ischaemic attack. Cerebrovasc Dis 2003;16 Suppl 1:14-19.

49. Heuschmann PU, Kolominsky-Rabas PL, Misselwitz B, Hermanek P, Leffmann C, Janzen RW, Rother J, Buecker-Nott HJ, Berger K: Predictors of in-hospital mortality and attributable risks of death after ischemic stroke: the German Stroke Registers Study Group. Arch Intern Med 2004;164:1761-1768.

50. Hassaballa H, Gorelick PB, West CP, Hansen MD, Adams HP, Jr.: Ischemic stroke outcome: racial differences in the trial of danaparoid in acute stroke (TOAST). Neurology 2001;57:691-697.
